# Allograft bone vs. bioactive glass in rehabilitation of canal wall-down surgery

**DOI:** 10.1038/s41598-023-44901-1

**Published:** 2023-10-20

**Authors:** Maxime Fieux, Romain Tournegros, Ruben Hermann, Stéphane Tringali

**Affiliations:** 1 Service d’ORLd’otoneurochirurgie et de chirurgie cervico-faciale, Centre Hospitalier Lyon Sud, Hospices Civils de Lyon, 69310 Pierre Bénite Cedex, France; 2 Université de Lyon, Université Lyon 1, 69003 Lyon, France; 3 UMR 5305, Laboratoire de Biologie Tissulaire et d’Ingénierie Thérapeutique, Institut de Biologie et Chimie des Protéines, CNRS/Université Claude Bernard Lyon 1, 7 Passage du Vercors, 69367 Lyon Cedex 07, France; 4 Service d’ORL et de chirurgie cervico-faciale, Hospices Civils de Lyon, Hôpital Edouard Herriot, 69003 Lyon, France

**Keywords:** Diseases, Medical research, Outcomes research

## Abstract

Canal wall-down (CWD) mastoidectomy creates a radical cavity that modifies the anatomy and physiology of the middle ear, thus preventing it from being self-cleaning and causing epidermal stagnation in the posterior cavities. Canal wall-down tympanomastoidectomy with reconstruction (CWDTwR) can obliterate such radical cavities. The main objective of this study was to compare postoperative results after CWDTwR by using either bone allografts or 45S5 bioactive glass as a filling tissue with an 18-month follow-up. This was a single-center observational trial including all patients undergoing CWDTwR. Patients were divided into two groups according to the filling material used: allograft bone (AB group) or 45S5 bioactive glass (BG group). Clinical monitoring was performed regularly, with control imaging performed at 18 months (CT scan and DW MRI). The two groups were compared with the t test for quantitative variables and the chi square test for qualitative variables (no revision surgery, audiometric results, complications, mastoid obliteration volume). Thirty-two patients underwent CWDTwR between October 2015 and 2018. The mean age was 48 years, and 71.9% (23/32) were men. A total of 46.9% (15/32) of the patients had undergone at least 3 middle-ear surgeries prior to CWDTwR. The most frequent preoperative symptom was otorrhea (100.0%, 32/32), and only 12.5% (4/32) experienced dizziness. Fifteen and 17 patients underwent surgery with bone allografts and 45S5 bioactive glass, respectively. At 18 months post-operation, 53.3% of the patients (8/15) in the AB group presented with recurrent otorrhea versus 5.9% (1/17) of patients in the BG group (p = 0.005). Seventy-eight percent (7/9) of symptomatic patients had undergone revision surgery at 18 months postoperation: 40.0% (6/15) in the AB group and 5.9% (1/17) in the BG group (p = 0.033). One patient’s surgery was cancelled due to the COVID-19 pandemic, and one patient refused surgery. The effects of CWDTwR with bone allografts are disappointing in early follow-up, with significant resorption leading to a 40.0% revision surgery rate. 45S5 BG is a simple solution, with preliminary results that are superior to those of AB. However, prospective controlled studies with longer follow-up times are needed to evaluate the value of BG versus other synthetic materials (such as hydroxyapatite) in surgical management of CWDTwR.

**Trial registration**: retrospectively registered.

## Introduction

Cholesteatoma is an aggressive disease that can lead to serious complications. It is well known that only surgical treatment can eradicate cholesteatoma and prevent its recurrence. To ensure complete removal of all of the epidermis that is present in the middle ear, resection of pathological tissue is typically accompanied by removal of healthy structures. Canal wall-down (CWD) mastoidectomy, which consists of removal of the external auditory canal posterior wall, allows for good visualization of the cavities, particularly the epitympanic region. In cases of major lysis of the bony framework, multiple recurrences requiring iterative surgery, or unfavorable anatomical conditions (such as a highly sclerotic mastoid, dural herniation, or a narrow corridor between the facial nerve and sigmoid sinus), a technique known as “canal wall-down tympanoplasty” enables obtaining low levels of residual cholesteatoma ranging from 0 to 13%^1^. Creation of such radical cavities modifies the anatomy and physiology of the middle ear, thus preventing it from being self-cleaning and causing epidermal stagnation in the posterior cavities. Additionally, the patient may be hindered during aquatic activities by direct thermal stimulation of the directly exposed lateral semicircular canal, which causes dizziness. Finally, conventional air hearing aids can be affected if the cavity is of a large size or in cases of recurrent otorrheic episodes^2^. To overcome these disadvantages, various surgical techniques have been developed to restore the primary anatomy of the external auditory canal by using the overlapping sliced cartilage autograft technique or mastoid and epitympanic filling techniques. Mosher introduced the concept of mastoid obliteration in 1911 when proposing creation of a flap from the back of the auricle^3^. Subsequently, many techniques have been proposed to reconstruct the posterior canal wall and to obliterate the mastoid and epitympanic cavities involving several materials, including biological tissue (muscle flaps, autografts with a mixture of bone dust and biological glue, or "bone pâté")^4,5^, as well as bone allografts; more recently, use of synthetic tissues, such as bioactive glasses, has been suggested^6–9^. Regarding use of bioactive glasses in middle ear surgery, several updates are important to consider for otologists: (i) several studies have been published on safety, anatomical and functional characteristics, and improvement of quality of life^10,11^; (ii) for some authors, bioactive glass appears to be the most reliable synthetic material in middle ear surgery^6^; (iii) performance in mastoid obliteration has now been established in the literature (with results showing that CWD revision surgery with mastoid obliteration and posterior canal wall reconstruction is superior to CWD revision surgery without mastoid obliteration in treatment of the troublesome mastoid cavity in both children and adults^12^); (iv) secondary mastoid obliteration is a safe and useful technique for treating the troublesome mastoid cavity in both children and adults (it is associated with a low cholesteatoma recidivism rate and a high rate of a trouble-free ear in the long term^13^); and (v) rates of recurrent and residual disease after CWU or CWD tympanoplasty with mastoid obliteration are shown to be qualitatively similar to, or better than, rates that have been previously reported for techniques without filling^14^. Canal wall-down tympanomastoidectomy with reconstruction (CWDTwR) enables reconstruction of the bony framework and filling of the cavities without the postoperative drawbacks of the CWD technique, as the framework is restored and supported by filling at end of procedure. This technique, which can be performed during the first operation for cholesteatoma, is useful regardless of the method employed to eradicate cholesteatoma^15^ and can safely be performed on an outpatient basis^16^.

The main objective of this study was to compare the efficacy of two materials used for mastoid filling in rehabilitation of radical cavities, bioactive glass 45S5 (BG, GlassBone^®^ Injectable Putty; Noraker, Lyon, France) and cancellous bone dust, referred to as allograft bone (AG) herein (AG, Phoenix^®^ chips 5–7 cc bone graft; TBF, Moins, France). The primary endpoint was the absence of new surgery at 18 months from all causes (persistent otorrhea or the presence of a residual cholesteatoma on MRI at 18 months). The secondary objectives included comparison of the postoperative course of hearing outcomes and the proportion of mastoids filled at 18 months. The last objective was based on the primary endpoint; our goal was to identify predictive factors of surgical success among selected preoperative variables.

## Methods

### Study design

The present study was conducted in a tertiary center. A retrospective chart review was performed for all patients who underwent reconstructive tympanoplasty after CWD mastoidectomy for cholesteatoma from July 2010 until January 2019. Although some patients had undergone reconstructive tympanoplasty on both sides, each patient was viewed as a single entity. This study complies with the ethical and legal requirements of French law (April 15, 2019) and the Declaration of Helsinki. This study was approved by an institutional review board (Agreement: 19–61), and oral informed consent from all participants was obtained.

### Characteristics of participants

Patient demographics, radiological evaluations, diagnoses, surgical details, complications, and clinical and audiological outcomes were collected. Inclusion criteria included all patients scheduled for CWDTwR after CWD mastoidectomy for cholesteatoma. Details regarding how to perform the surgical technique have already been published^17^. Exclusion criteria included patients who had undergone CWD mastoidectomy for any reason other than cholesteatoma, subjects with other middle ear diseases, and patients with less than 18 months of follow-up. All patients received standard preoperative assessment, including history, physical examination, and pure-tone audiometry (PTA), consisting of both air conduction (AC) and bone conduction (BC) threshold measurements inside a standard soundproof room before the intervention. Following the recommendations of the committee for Hearing and Equilibrium of the American Academy of Otolaryngology—Head and Neck Surgery, audiological data were collected to reduce hearing measurement variability between studies^9^. All patients underwent PTA tests from 250 Hz to 4 kHz as well as at 8 kHz to measure high-frequency hearing preservation. The means of the thresholds for bone and air conduction at 0.5, 1, 2, and 4 kHz were used to form a four-tone pure-tone average; as the 3 kHz frequency is not evaluated in our center, the mean of the 2 and 4 kHz frequencies was considered for the 3 kHz frequency. The functional results were evaluated by comparing the preoperative and 1-year postoperative bone conduction threshold level averages.

### Interventions and comparisons

All patients were evaluated radiologically at 18 months postoperatively using a high-resolution computed tomography (HRCT) scan of the temporal bone and MRI to measure the volume and location of the mastoid and epitympanic obliteration and any possible signs of complications or residual cholesteatoma in the middle ear. Temporal bone HRCT acquisition was performed using a Discovery 750HD CT scanner (GE Healthcare, Chicago, USA) with the following parameters: a collimator of 0.625, with reconstruction with 0.6 mm slice thickness at 0.4 mm intervals. MRI studies were performed with a 3-T MR device (HDxT, GE Healthcare) with an eight-channel head coil. MRI acquisition was centered around the temporal bone and consisted of axial T1-weighted (T1-w) spin echo imaging (slice thickness 1.5 mm), high-resolution 3D T2-weighted (T2-w) imaging (slice thickness 0.4 mm), axial and coronal nonecho planar (EP) diffusion-weighted (DW) imaging (slice thickness 3 mm), and a 3D spoiled gradient-recalled T1-w fat-saturated sequence acquired after gadolinium injection (0.2 mL/kg, Dotarem). HRCT images were reconstructed for each ear in the plane of the lateral semicircular canal. IntelliSpace Portal software (Medisys, Philips Research, Suresnes, France) was used for 3D semiautomated quantitative assessment of mastoid obliteration at baseline (18 months postoperatively). First, an experienced otolaryngologist segmented the mastoid volume in 3D using an interactive mouse “click and drag” function within the lesion on HRCT axial slices. With non-Euclidean radial basis functions, a 3D segmentation mask was generated and edited in the same way for all slices. With the 3D segmentation mask and the image matrix dimensions and slice thickness, the software calculates the volume (in cm^3^)^18–20^. All quantitative and qualitative image analyses were then performed by two experienced radiologists, and a consensus between the raters was used in cases of conflicting evaluations.

Surgeries were performed by one senior surgeon who was an expert in this surgery to ensure as much similarity in the surgical approaches as possible and to reduce the impacts of the learning curve and operator skill differences. All of the patients for whom other surgeons performed this procedure were excluded from the study (5 interventions). All surgeries were performed with the patients under general anesthesia and included the NIM-Response 3.0 facial nerve monitoring system (Medtronic, Jacksonville, FL, USA). A retroauricular skin incision was made, and a musculoperiosteal flap was created. Perichondrium and cartilage grafts were harvested from the cymba and cavum conchae to compensate for the posterior canal wall defect of the external acoustic meatus and to cover the entire filled zone. After complete removal of the cholesteatoma, reconstruction was started in the middle ear, including ossiculoplasty and tympanic membrane grafting. CWDTwR was conducted using a large cartilage graft with perichondrium to reconstruct the posterior canal wall. The last step before suturing involved epitympanic and mastoid cavity obliteration by using either BG or AG (AG was used before BG, which was available 12 to 20 months later). It was important that the BG was perfectly isolated from the external acoustic meatus by carefully covering all of the cartilage with perichondrium (details regarding the procedure have been previously published^17^). Careful suturing of the musculo-periosteal flap and the retroauricular incision with absorbable sutures was necessary to prevent dissemination of the implanted BG. All patients received perioperative antibiotic prophylaxis with amoxicillin/clavulanic acid, which was continued for 7 days postoperatively (pristinamycin in the case of allergy to amoxicillin). Except for patients with heavy medical comorbidities or for social reasons, most procedures were conducted as a one-day surgery in the ambulatory setting.

### Statistical analysis

The need for revision surgery was the primary criterion for defining BG obliteration efficacy. Surgical revision was necessary for two main reasons: (i) recurrent otorrhea or difficulty in fitting the hearing aid and (ii) enclosure of the cholesteatoma within the obliterated cavities or residual disease on 18-month postoperative MRI. Residual cholesteatoma was evaluated on 18-month postoperative MRI. A positive functional impact on hearing was defined as a postoperative ABG of strictly less than 20 dB. Categorical variables are summarized as frequencies and percentages, and continuous variables are described as means and standard errors. At diagnosis, characteristic comparisons between groups were assessed with the chi square test for categorical variables and the independent two-sample t test for continuous variables. An odds ratio coefficient with its 95% confidence interval (CI) was calculated for each characteristic included in a multivariate logistic regression model to identify predictive factors of surgical success. Surgical success was defined as the absence of surgical revision. Variables were included if they were known to have a strong influence on postoperative healing or if their p values were > 0.20 after univariate logistic regression analysis. Results were considered statistically significant at p value ≤ 0.05. Statistical analyses were performed with R version 3.5.3 (R Foundation for Statistical Computing, Vienna, Austria).

### Ethics approval and consent to participate

This study complies with the ethical and legal requirements of French law (April 15, 2019) and the Declaration of Helsinki. This study was approved by the institutional review board “Comité d’Ethique du CHU de Lyon” (Agreement: 19-61). Oral informed consent to participate was obtained from all of the participants.

## Results

### Preoperative demographic data

Thirty-two patients were included according to the inclusion criteria. The mean age was 47 ± 15 years; there were 23 males and 9 females, 18 right ears and 14 left ears. A total of 46.9% (15/32) of the patients had undergone at least 2 tympanoplasties before CWD mastoidectomy for creation of a radical cavity and to ascertain definitive eradication of cholesteatoma (3rd operation). All patients had otorrhea at management, but only 12.5% (4/32) had vertigo. Conventional hearing aid fitting was considered difficult for 11 of the 68.8% (22/32) of patients who needed one. There were 10 patients whose residual hearing after one or more tympanoplasties was sufficiently good and thus did not need any hearing aids. A total of 62.5% (20/32) of the cohort had undergone CWD mastoidectomy before 2010, and the remaining 37.5% (12/32) of patients underwent the procedure between 2010 and 2019. The average overall patient follow-up time was 15 ± 9 years. The patients were divided into two groups to compare the efficacy of the filling materials: BG was used for 51.5% (17/32) of the patients, comprising the BG group, whereas cancellous bone meal was used for 46.9% (15/32), comprising the AB group. The demographic, audiometric and clinical characteristics of the patients by group are detailed in Table 1. The mean postoperative follow-up time differed significantly between the BG group (33 ± 8 months) and the AB group (44 ± 13 months) because their use was sequential (AB was first used; BG was used later, when available). No other significant differences were found preoperatively between the two groups; in other words, the patients were considered to be comparable with regard to age, sex, otologic and medical history, initial clinical presentation, and audiometric status for the remainder of the statistical analyses.

**Table 1:** Patient demographic data, preoperative presentations and operated side.

N (%)	BG Group	AB Group	p value
	n = 17 (51.5%)	n = 15 (46.9%)	
Age (years)	51 ± 19	44 ± 10	0.142
Sex			
Male	11 (64.7%)	12 (80.0%)	0.444
Female	6 (35.3%)	3 (20.0%)	
Operated side			
Right	9 (52.9%)	9 (60.0%)	0.964
Left	8 (47.1%)	6 (40.0%)	
Otologic history (years)	15 ± 9	14 ± 9	0.97
Number of interventions			
1 or 2	9 (52.9%)	6 (40.0%)	0.383
≥ 3	8 (47.1%)	9 (60.0%)	
Date of CWD surgery			
< 2010	11 (64.7%)	9 (60.0%)	1
≥ 2010	6 (35.3%)	6 (40.0%)	
Medical history			
None	14 (82.4%)	13 (86.7%)	0.486
Facial palsy	0 (0.0%)	1 (6.7%)	
TBF failure	3 (17.6%)	0 (0.0%)	
T21	0 (0.0%)	1 (6.7%)	
Preoperative clinical symptoms			
Otorrhea	15 (88.2%)	13 (86.7%)	1
Vertigo	2 (11.8%)	2 (13.3%)	
PTA air conduction threshold (dB)	52 ± 13	57 ± 27	0.94
PTA bone conduction threshold (dB)	25 ± 10	31 ± 35	0.416
PTA-ABG (dB)	26 ± 11	26 ± 14	0.985
Postoperative follow-up (months)	32 ± 8	44 ± 13	< 0.006*

Quantitative variables are shown as the mean (standard error), and qualitative variables are shown as the total population (percentage). Surgery corresponds to the date of canal wall reconstruction tympanoplasty. *Corresponds to statistical significance (p value < 0.05). Pure-tone average (PTA) air and bone conduction thresholds were computed as the average thresholds at 0.5, 1, 2, and 3 kHz. PTA air bone gap (PTA-ABG) closure was computed as the difference between PTA air and bone conduction. CWD surgery: canal wall-down surgery.

### Postoperative clinical outcomes at 18 months

Eighteen months after reconstructive surgery, 40.0% (6/15) of the patients in the AB group and 1 patient in the BG group had undergone revision surgery (p = 0.033). Of the 6 patients who needed revision surgery in the AB group, 4 presented with persistent otorrhea and material extrusion and 2 with residual cholesteatoma. The patient in the BG group needed revision surgery for residual cholesteatoma. Another patient presented with material extrusion from the retroauricular scar, but local care was sufficient to heal the wound; therefore, no surgery was performed. In the AB group, 26.7% (4/15) of the patients presented with otorrhea but did not need revision surgery. A total of 88.2% (15/17) and 33.3% (5/15) of patients in the BG and AB groups, respectively, were satisfied with the radical cavity reconstruction (no otorrhea, no residual cholesteatoma or revision surgery needed and good aquatic tolerance) (p = 0.005). The detailed results are shown in Table 2.

**Table 2:** Postoperative clinical and radiological results.

	BG Group	AB Group	p value
	n = 17 (51.5%)	n = 15 (46.9%)	
Otoscopy			0.005*
Healing complete	15 (88.2%)	5 (33.3%)	
Otorrhea	1 (5.9%)	4 (26.7%)	
Extrusion rate	1 (5.9%)	6 (40.0%)	
ABG closure (< 20 dB)	12 (70.6%)	10 (66.7%)	1
Conventional hearing aid			0.001*
Not necessary	7 (41.2%)	3 (20.0%)	
Good fitting	9 (52.9%)	2 (13.3%)	
Failed fitting	1 (5.9%)	10 (66.7%)	
Time before CT scan	20 ± 3	21 ± 5	0.806
CT scan assessment at 18 months			
Bone attenuation measurements (HU)	870.15 ± 45	555.05 ± 138	< 0.001*
Total mastoid volume (cm^3^)	2.09 ± 0.6	2.10 ± 0.8	0.850
Obliteration volume of the mastoid (cm^3^)	1.62 ± 0.83	0.63 ± 0.30	0.002*
Mastoid cavity obliteration (%)	76.40 ± 25	34.44 ± 30	0.001*
Successful epitympanic obliteration	15 (88.2%)	2 (13.3%)	< 0.001*

Quantitative variables are shown as the mean (standard error). * corresponds to statistical significance (p value < 0.05).

### Hearing results

Before CWDTwR, 25% of the patients had slight hearing loss (PTA air conduction threshold < 40 dB), 50% had mild to moderate hearing loss (PTA air conduction threshold between 40 and 70 dB), and 25% had severe to profound hearing loss (PTA air conduction threshold > 70 dB). The mean preoperative PTA air conduction threshold was 53 ± 21 dB, and there was no significant difference between the BG (52 ± 13 dB) and AB (57 ± 27) groups (p = 0.940). The same results were found regarding the PTA bone conduction threshold and the PTA-ABG between the two groups, as detailed in Table 1. The mean PTA bone conduction thresholds were 31 dB and 25 dB preoperatively and 32 dB and 28 dB postoperatively in the AB and BG groups, respectively. The mean PTA air conduction thresholds were 57 dB and 52 dB preoperatively and 46 dB and 44 dB postoperatively in the AB and BG groups, respectively. Differences between the pre- and postoperative values were not significant for either bone or air conduction audiometry, with p values of 0.454 and 0.663, respectively. Air–bone gap closures were 12 dB and 11 dB in the AB and BG groups, respectively, with no significant difference between the two groups, as detailed in Table 2.

### Radiological obliteration

**Figure 1:**
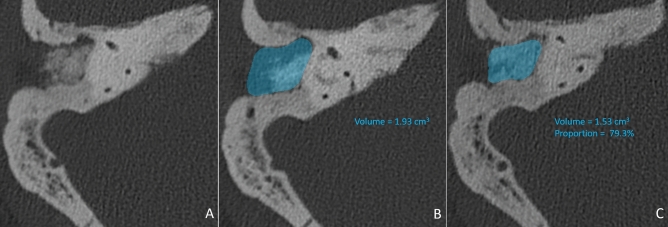
(**A**) Postoperative right ear high-resolution computed tomography (HRCT) scan of the temporal bone at 18 months postoperation to show how volumetric measurements were performed. HRCT images were reconstructed for each ear in the plane of the lateral semicircular canal. (**B**) Mastoid volume assessment at baseline (18 months postoperation, blue). IntelliSpace Portal software (Medisys, Philips Research, Suresnes, France) was used for the 3D semiautomated quantitative assessment of volume. It was segmented in 3D by using an interactive mouse “click and drag” function within the lesion on HRCT axial slices, after which a 3D segmentation mask was generated and edited in the same way for all slices. With the 3D segmentation mask and the image matrix dimensions and slice thickness, the software calculated the volume (in cm^3^). (**C**) Mastoid obliteration volume assessment at 18 months postoperation (blue). The same software (Intellispace portal) was used for 3D semiautomated quantitative assessment of mastoid obliteration volume.

Bone attenuation measurements in Hounsfield units (HU) in the region of interest of the obliteration material were significantly different between the two groups (p < 0.001). Indeed, the mean bone attenuation in the AB group was 555.05 ± 138, versus 870.15 ± 45 in the BG group. Mastoid volume measurements were similar in both groups: 2.10 cm^3^ and 2.09 cm^3^ in the AB and BG groups, respectively (p = 0.850). On the other hand, there was a significant difference in the proportion of mastoid cavity obliteration between the AB and BG groups (34% ± 30 vs. 76% ± 25, p = 0.001). Details of the volume measurements are shown in Fig. 1. Regarding the precise localization of the obliteration, successful epitympanic obliteration was achieved in 88.2% (15/17) of the patients in the BG group and 13.3% (2/15) in the AB group (p < 0.001). No middle ear opacity was found on HRCT images in either group. MR images were analyzed to identify residual cholesteatoma; 2 were identified in the AB group and 1 in the BG group, but the difference was nonsignificant. Details are shown in Table 2.

### Predictive factors of surgical success

**Figure 2:**
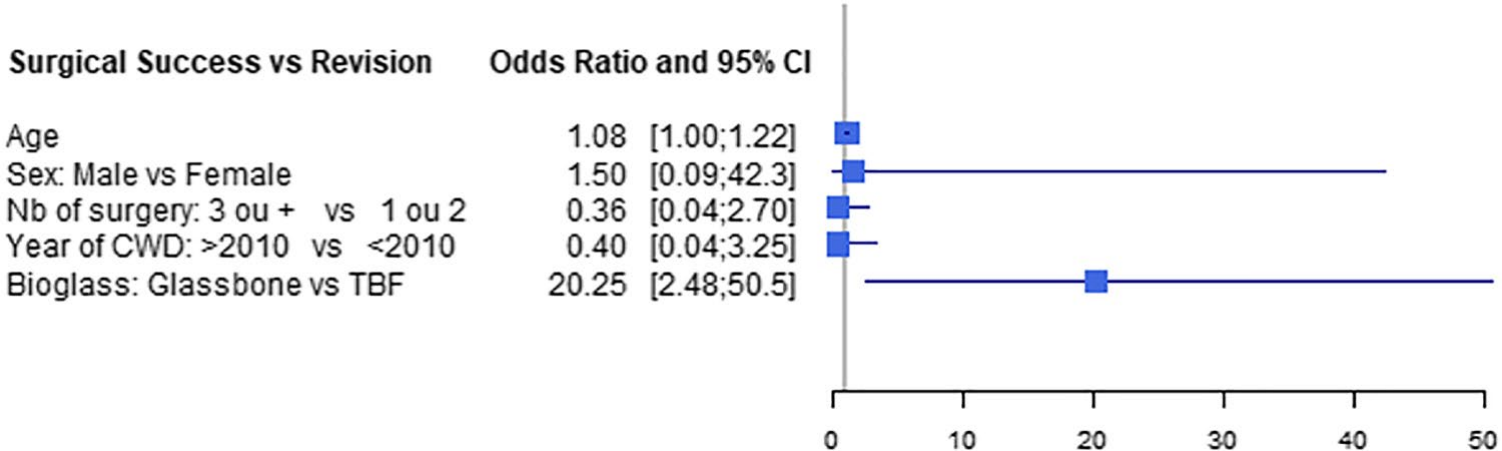
Forest plot of estimated coefficients from multivariate logistic regression. Results are reported as odds ratios with their 95% confidence intervals.

The primary criterion for surgical success was lack of a need for revision surgery at 18 months regardless of the motivation (residual cholesteatoma or persistent otorrhea). Multivariate logistic regression was performed after adjusting for age, sex, number of previous surgeries, year of CWD mastoidectomy, and the type of obliteration material used. The only relevant factor significantly predictive of the absence of revision surgery at 18 months was use of BG as the obliteration material (OR = 20.25, 95% CI [2.481–50.547]; p = 0.016). For the number of surgeries, two or fewer was taken as a reference. Usually, the need for 3 or more surgeries for cholesteatoma eradication is associated with highly aggressive disease. The reference categories chosen for the year of CWD mastoidectomy and for sex were between 2000 and 2010 and female, respectively. None of these variables had any impact on the estimated OR, indicating a stable model. The multivariate logistic regression results are shown in Table 3. The odds ratios and 95% confidence intervals for selected variables are also shown in a forest plot in Fig. 2.

**Table 3:** Multivariable logistic regression model for predictive factors of postoperative success.

	Coef	95% CI LL	95% CI UL	p value
Age	1.085	0.997	1.218	0.096
Sex				
Female	Reference			
Male	1.505	0.089	42.318	0.778
Number of surgeries				
1 or 2	Reference			
3 or more	0.361	0.037	2.697	0.334
Year of CWD mastoidectomy				
Before 2010	Reference			
2010–2018	0.395	0.037	3.249	0.4
Obliteration material	Reference			
Cancellous bone meal	20.245			
45S5 bioactive glass		2.481	50.547	0.016*

Postoperative success (no surgery at 18 months) was estimated through a multivariable logistic analysis to identify explanatory variables. Estimated coefficients are adjusted for confounding factors. Results are shown with 95% confidence intervals (95% CIs). If the 95% CI excludes 0, the results are statistically significant, represented by * (p value < 0.05).

Coef: estimated coefficient; 95% CI: 95% confidence interval; LL: lower limit; UL: upper limit.

## Discussion

Forty percent (6/15) of patients in the AB group and one patient in the BG group presented with persistent otorrhea or residual cholesteatoma that needed revision surgery. There was no statistical significance found between the pre- and postoperative bone or air conduction audiometry thresholds or for ABG closure between the two groups. A total of 88.2% (15/17) and 33.3% (5/15) of the patients were satisfied with the radical cavity reconstruction in the BG and AB groups, respectively. The only significant predictive factor of surgical success was use of 45S5 bioactive glass relative to bone allografts after adjusting for age, sex, year of CWD mastoidectomy and number of previous surgeries.

The interest and reliability of the rehabilitation technique evaluated in this study has resulted in development of various obliteration techniques immediately in the first step of the management of cholesteatoma. The “remove and rest” technique with mastoid obliteration, as described by Gantz^8^, allows for obliteration of the mastoid cavity on the first-stage surgery for cholesteatoma. It offers wide exposure for cholesteatoma resection (when the bony framework of the external auditory meatus is removed), without the discomfort of a radical cavity after a classic CWD procedure (because the framework supported by the obliteration material is restored). Another study^21^ showed no significant difference in quality of life between CWD and CWU surgery, which was confirmed by an even more recent study^22^.

Currently, the advantages and disadvantages of the obliteration materials chosen (biological versus synthetic) remain controversial. Biological materials (muscle, fascia, or fat) from the patient are sometimes lacking because of previous surgery^6,23^. Advantages of biological materials focus on financial cost; however, one disadvantage is that the material tends to retract over time^6^. Bone parenchyma^24^ is similar to human bone and induces local osteogenesis. However, its use remains controversial due to the high rate of postoperative infection and its high resorption rate. This resorption leads to recurrence of tympanic membrane atelectasis due to bony defects in the posterior wall of the external acoustic meatus. The long-term failure rate is reported to be greater than 50%^6,24^. Nevertheless, some authors have shown that the rate of postoperative infection can be significantly reduced with peri- and postoperative administration of antibiotics^25^. In this study, there was a discrepancy in the follow-up of patients (44 months versus 32 months for the AB and BG groups, respectively) because AB was the first obliteration material that was available, 12 months prior to BG. Symptoms (mainly otorrhea) recurred after the first month in favor of very rapid resorption of the material, which was the reason for the modification of our choice of material and not of surgical technique.

To solve these problems, many synthetic materials, such as hydroxyapatite, two-phase calcium phosphate, bioactive glass, titanium, and silicone, are now available^6,26^. These materials are easy to use and immediately available in great quantities without the need for an additional patient sampling site (for example, the contralateral ear), which would extend the operating time^26^. However, use of new synthetic obliteration devices still requires close clinical and radiological monitoring. For example, SerenoCem has been reported to cause secondary resorption of bone adjacent to the implantation site, thus leading to its withdrawal from the market^27^.

The efficacy of mastoid obliteration is based on the depiction of a new external auditory meatus in the correct position on postoperative otoscopy, which can also identify complications such as infection or extrusion of the material. Nevertheless, several studies have shown good results with use of BG, with an overall extrusion rate of less than 0.5%^6^. In 96% of cases, the ear is dried out^6,9,28^. Although hydroxyapatite remains the second most commonly used synthetic material for mastoid obliteration, its use does not seem to produce stable results in the long term. Indeed, unlike BG, the overall incidence of extrusion is reported to be greater than 15%^6^, but these results are not consistent^6^. According to the author’s experience, BG might have a better outcome than hydroxyapatite because of better osteointegration and antibacterial properties leading to less infection and extrusion, therefore enabling restoration of a self-cleaning ear once healed. Among the weaknesses of this study, one limitation includes the number of patients. Indeed, the sample size was small because of the highly focused nature of the surgical procedure and the patient population of a single surgical center. However, a single surgical center was needed to homogenize the surgical procedure. Additionally, this study was retrospective. Obviously, a retrospective review is less satisfactory than a prospective randomized study, but surgeries were initially performed with AB and then with the BG technique due to the disappointing results of recurrent otorrhea in the first group and the availability of the materials. Furthermore, there was a relatively short follow-up time, which may explain the very low residual cholesteatoma rate (2/15 for the AB group and 1/17 for the BG group), even though the follow-up time was sufficient to estimate recurrence or residual cholesteatoma. There was a recent review of the literature that aimed to compare data between single-stage tympanoplasty and mastoid obliteration for acquired cholesteatoma. Although it is difficult to compare different studies, it was shown that the rate of recurrent and residual disease after use of single-stage mastoid obliteration with either CWU or CWD tympanoplasty is qualitatively similar to, if not better than, rates that have been reported for nonobliterative techniques. However, it should be noted that although surgeon preference and patient factors remain determinants in the choice of surgical technique, CWU tympanoplasty with obliteration may be preferred to CWD tympanoplasty with obliteration because recurrence of infection is lower^14^. Canal wall reconstruction techniques have several drawbacks (risk of residual cholesteatoma and of recurrence of cholesteatoma if canal-tympanic continuity is not perfectly restored). On the other hand, quality of life after CWD surgery is known to be poorer than after CWU surgery due to creation of a large recess cavity, as previously described. Indeed, it requires more instrumental procedures due to cerumen accumulation in the posterior cavities; precautions must be taken when swimming, as contact with cold water can induce dizziness, making use of air-conduction hearing aids more difficult. Rehabilitation techniques after CWD surgery (CWDTwR), such as that described in this study or immediately during the first procedure [canal wall reconstruction (CWR) tympanoplasty or canal wall up-down^17^], enhance quality of life (less dizziness and otorrhea)^21,22^. All this limits resorting to ENT consultation^24^ and facilitates use of air-conduction hearing aids^22^.

## Conclusion

Canal wall-down mastoidectomy rehabilitation with BG mastoid obliteration can significantly improve the quality of life of patients who undergo multiple operations without increasing risk of recurrent or residual cholesteatoma, provided that the surgical technique is perfectly mastered. Furthermore, BG appears to be more valuable than cancellous bone meal. However, prospective controlled studies with longer follow-up are needed to evaluate the value of BG versus other synthetic materials (such as hydroxyapatite) in surgical management of CWDTwR.

## Data Availability

The datasets used and/or analyzed during the current study are available from the corresponding author upon reasonable request. All of the methods were conducted in accordance with relevant guidelines and regulations.

## Abbreviations

AC: Air conduction
ABG: Air–bone gap
AB: Allograft bone
BC: Bone conduction
BG: Bioactive glass
CT: Computed tomography
CWD: Canal wall down
CWDTWR: Canal wall-down tympanomastoidectomy with reconstruction
CWU: Canal wall up
DW: Diffusion weighted
ENT: Ear nose and throat
HRCT: High-resolution computed tomography
HU: Hounsfield units
MR: Magnetic resonance
MRI: Magnetic resonance imaging
OR: Odds ratio
PTA: Pure-tone audiometry
T1-w: T1-weighted
T2-w: T2-weighted
